# The Inoculation of Tubercle

**Published:** 1867-11

**Authors:** 


### The Inoculation of Tubercle.

One of the most striking and suggestive pieces of work done lately in the region of scientific medicine is that effected by M. Villemin with a view to ascertain the inoculabllity of tubercle. The tubercular class of diseases is one of the largest in the nosology of the Registrar-General. It causes the death of some 60,000 persons a year. It is not inferior in importance to the zymotic class. It is superior. For whereas zymotic diseases kill about the same number of persons as the tubercular class, there is something in the tubercular constitution which determines largely the fatality and seriousness of many of the zymotic diseases, and yet, although the tubercular diathesis is so important and so fatal, it is remarkable that we know so little, and have done so little, in the investigation of tubercular disease. We have grand special hospitals for the treatment of the tubercular affections, especially for the treatment of phthisis. The disease is so common, so disabling, and so little amenable to treatment in a general hospital, that the prevalent objections to special hospitals have not been much urged against these. But have they done all that might reasonably have been expected of them in the elucidation of the origin, the nature, and the laws of tubercular disease? Scarcely, we think. Neither the pathology of the disease nor its treatment has received much development from the special hospitals. It is from France chiefly that our knowledge of tubercle has come, and it is to France that we are indebted for the last great conception on the subject, which enlarges and excites all our ideas on it, and which seems destined to be verified by physiological experiment.

It is not our intention to dwell at any length on the experiments of M. Villemin. We thought them so important as to give an abstract of his own account of them. Onr present design is rather to direct particular attention to the experiments of later observers, which strikingly agree with those of M. Villemin. Many English pathologists of the greatest weight have repeated his experiments, and, with perhaps one important exception, that of Dr. Andrew Clark, have got similar results, and arrived at similar conclusions. But the most important confirmation of the substantial accuracy of M. Villemin's conclusions is to be found in the Report of a Commission of the Academy of Medicine on the two memoirs of M. Villemin, which we noticed in the July and August numbers, and which is now before us in *La France Medicale* of the 20th of July. This Commission was composed of MM. Louis, Grisolle, Bouley, and Colin. M. Colin read the Report in the name of the Commission, and included in it an account of several experiments by the Commission, which yielded results essentially similar to those obtained by M. Villemin. At the very outset of the Report there occur words which indicate the opinion of the members of this Commission on the main conclusions of M. Villemin. They give him credit for throwing light by physiological experiment on medicine, and say that the two memoirs presented by him to the Academy, on the 5th of December, 1865, and on the 30th of November last, reveal to us a fact of the highest degree—the transmission of phthisis by the inoculation of tuberculous matter.

Their endorsement of M. Villemin's main conclusions is all the more effective from the fact that they do not hesitate to say that in one or two minor points he has come to hasty or incorrect conclusions. Two of these are in particular pointed out. First, they aver that M. Villemin was inexact in believing that sheep were insusceptible of tuberculosis, and that he too quickly concluded that the tubercles in the cow and those in man were of the same nature. Mr. Colin, as we have said, gives in the Report an account of the various experiments with the inoculation of tubercle by the Commission. Some of these failed; but the most of them succeeded perfectly. The failures were suggestive to M. Colin. He procured from M. Villemin a specimen of the tuberculous matter used by him. This included fragments of various kinds of tuberculous matter, old and recent, transparent and grey, firm and softened. He reduced all into a homogeneous pulp, and inserted portions of this into four rabbits at the base of the ear. Only one of these animals became affected with tuberculosis. M. Colin accounts for the failure principally by the fact that in examining two of the animals he found that the tuberculous matter inserted had become encysted at the seat of wound, and so had become protected from absorption. In his subsequent experiments he was careful to go deeper, and to spread the matter over a larger surface, and so he obtained success. These later experiments are valuable, not only as additioual to M. Villemin's, but as made with every distinct form of tubercle used separately. Fine miliary tubercle, softened caseous matter, hard tubercle taken from an ox affected with the calareous forms of phthisis, yellowish tubercle in course of the so-called regressive metamorphosis, and lastly, slices of a tumor full of strongles taken from a sheep affected with verminous phthisis, were all used, and all with similar results. We shall give as a specimen M. Colin's account of his first experiment. It illustrates not only the phthisical result obtained, but the effects produced in nearly all M. Colin's experiments on the lymphatic vessels and glands, and upon which he founds important conclusions:

"A rabbit was inoculated with fine miliary granulations taken from a cow. He died, with all the appearances of phthisis, after two months and some days. The lungs were strewed with tubercles; the liver, the spleen, and one of the kidneys presented tubercles; the glands of the neck and of the ear were swollen. Finally, from the point where inoculation had been effected there proceeded white tracks, like farcinous cords, [des trainées blanches, semlable à des cordes farcineuses]."

The glandular results are thus described in the second experiment. The rabbit had become tuberculized after the inoculation with softened caseous tubercular matter:

"The inguinal glands, the axiliary, the prepectoral on the side of inoculation, were hypertrophied, and penetrated with matter of caseous aspect."

The principal conclusion to which the various experiments led is thus stated in the report: "Thus in all the degrees of its evolution, and in all its forms, tubercle comports itself in an identical manner."

The time has not yet come for anything more than the examination of facts on this subject. One immediate effect of the facts already accumulated will be to give great additional value to the study of the localities of tubercle as met with in the morbid conditions of the human system, not only of the more recent and palpable seats of it, but of the glands, cervical and mesenteric, in which it is apt to be developed in the earlier years of life. Whatever may be the fact as to the inoculability of tubercle, it is hard to understand how it can explain the occurrence of the ordinary forms of the disease which we see, whether we believe with M. Villemin in a period of inoculation and a local reproduction of inoculated matter, or with M. Colin in the removal of it to the lungs and other central organs, there to provoke a secondary eruption. M. Colin doubts whether the lung in the adult is always primarily affected, even when it appears to be so. He thinks it may be that the phthisis results from the dissolution and displacement of tubercular matters deposited during youth in the glands and other important organs, and remaining dormant in them for a greater or less amount of time. This hypothesis is very ingenious, but not very probable. It is equally difficult to understand, on M. Colin's theory, how tubercle deposited in glands in early life should not be transferred while yet soft and fresh to central parts; and how, after the lapse of years, when the more inoculable parts of it have probably been removed by absorption, it should be carried to distant parts and there become active. But these difficulties in no way detract from the value of the experiments and the report of M. Colin and his fellow commissioners.

An interesting question is, the extent to which the contagious or inoculate character of tubercle is possessed by it exclusively. The experiments of the Commission show that a great variety of substances, under the generic name of "tubercle," have the quality of reproducing themselves in the central organs; and they go to show that all inflammatory products have a similar tendency, including pus. The exact relation of tubercle to inflammatory deposits is yet a moot point amongst pathologists. All that can be said here is, that experiment shows that there is a considerable similarity between the deportment of tubercle and other inflammatory products when inoculated. According to M. Colin, the other inflammatory products act in the same direction, but not to the same extent, as the grey grauulation. "Les produits morbides présentés comme des resultats d'inflammation on de regression n'agissent pourtant pas autant que la granulation grise."—*Lancet.*

